# The Preserflo MicroShunt Affects Microvascular Flow Density in Optical Coherence Tomography Angiography

**DOI:** 10.3390/biomedicines11123254

**Published:** 2023-12-08

**Authors:** Jens Julian Storp, Hannah Schatten, Friederike Elisabeth Vietmeier, Ralph-Laurent Merté, Larissa Lahme, Julian Alexander Zimmermann, Verena Anna Englmaier, Nicole Eter, Viktoria Constanze Brücher

**Affiliations:** 1 Department of Ophthalmology, University of Muenster Medical Center, 48149 Muenster, Germanyfriederikeelisabeth.vietmeier@ukmuenster.de (F.E.V.); ralph-laurent.merte@ukmuenster.de (R.-L.M.); larissa.lahme@ukmuenster.de (L.L.); julian.zimmermann@ukmuenster.de (J.A.Z.); verenaanna.englmaier@ukmuenster.de (V.A.E.); nicole.eter@ukmuenster.de (N.E.); viktoria.bruecher@ukmuenster.de (V.C.B.); 2 Augenklinik Roth am St. Josef-Hospital, 53225 Bonn, Germany

**Keywords:** surgery, device, intraocular pressure, IOP, vessel density, MIGS, LIGS, glaucoma, severity

## Abstract

Intraocular pressure (IOP) lowering surgery has been shown to alter microvascular density in glaucoma patients. The aim of this study is to report changes in retinal flow density (FD) over the course of treatment with the Preserflo MicroShunt, using optical coherence tomography angiography (OCTA). 34 eyes from 34 patients who underwent Preserflo MicroShunt implantation were prospectively enrolled in this study. OCTA imaging was conducted at the superficial (SCP), deep (DCP) and radial peripapillary plexus (RPC) levels. The progression of FD and IOP was assessed at different time points from baseline to six months postoperatively for the entire patient population, as well as disease severity subgroups. The Preserflo MicroShunt achieved a significant reduction in IOP over the course of six months (median: 8 mmHg; *p* < 0.01). FD values of the SCP and DCP did not show significant fluctuations, even after adjusting for disease severity. FD of the RPC decreased significantly over the course of six months postoperatively from 42.31 at baseline to 39.59 at six months postoperatively (*p* < 0.01). The decrease in peripapillary FD was strongest in patients with advanced glaucoma (median: −3.58). These observations hint towards dysfunctional autoregulatory mechanisms in capillaries surrounding the optic nerve head in advanced glaucoma. In comparison, the microvascular structure of the macula appeared more resilient to changes in IOP.

## 1. Introduction

Glaucoma is a global leading cause of blindness [1]. The “mechanical theory” of glaucoma progression regards intraocular pressure (IOP) as one of the main risk factors associated with the disease [2]. The majority of therapeutic interventions therefore target IOP reduction, which can be achieved by topical medication, non-penetrating procedures, or penetrating surgery. In the recent past, the need to reduce the postoperative care burden has given rise to new microinvasive approaches [3], which have been coined “MIGS” (microinvasive glaucoma surgery) or “LIGS” (less invasive glaucoma surgery). The Preserflo MicroShunt (Santen, Miami, FL, USA), an 8.5 mm long tubular structure with a 350 µm outer diameter and 70 µm lumen made from biocompatible (poly)styrene-block-isobutylene-block-styrene, represents a novel LIGS device and has been shown to effectively lower IOP in glaucoma patients [3,4,5,6,7].

The “mechanical theory”, however, falls short in explaining the occurrence of normal-tension glaucoma and the fact that in some patients, simply lowering IOP does not halt the progression of the disease [1,8]. In response, the “vascular theory” of glaucoma development was proposed, according to which glaucoma is considered to result from inadequate blood supply to the eye. According to this theory, the development of glaucoma cannot be attributed solely to increased IOP but also to changes in systemic and microvascular perfusion [9,10].

Studies using optical coherence tomography angiography (OCTA), a new imaging technology that allows for the quantification of retinal microvasculature [11], have identified distinct changes in in glaucoma patients [12,13,14,15,16,17,18] and after IOP lowering surgery [19,20,21,22,23,24]. Bridging the gap between mechanical and vascular theories, these studies highlight the need for a comprehensive understanding of microvascular alterations attributed to less invasive glaucoma surgeries, particularly those involving devices like the Preserflo MicroShunt. Our study seeks to contribute to fill this gap in knowledge by quantifying longitudinal changes in retinal microvascular parameters associated with Preserflo MicroShunt implantation in glaucoma patients. Through this exploration, we aim to provide valuable insights into the microvascular dynamics influenced by this less invasive surgical approach, furthering our understanding of glaucoma management beyond the conventional mechanical paradigm.

This study aims to quantify longitudinal changes in retinal microvascular parameters associated with implantation of the Preserflo MicroShunt in glaucoma patients. 

## 2. Materials and Methods

### 2.1. Design and Setting

All procedures were carried out in compliance with the 1964 Helsinki Declaration and its later amendments. This study was approved by the ethics committee of the Medical Association of Westfalen-Lippe and the University of Münster (No.: 2015-402-f-S). Data for this monocentric trial were collected from glaucoma patients visiting the Department of Ophthalmology at the University Hospital Münster, Germany, between August 2020 and November 2022. General patient information was retrieved from electronic patient files stored in the digital documentation system FIDUS (Arztservice Wente GmbH, Darmstadt, Germany).

### 2.2. Patient Examination

All patients older than 18 years of age, who were scheduled to receive Preserflo MicroShunt implantation due to glaucoma, were eligible for study inclusion. The following inclusion criteria were used: confirmed diagnosis of glaucoma, and the absence of any further retinal or neurological diseases. Individuals who had media opacities that precluded high-quality imaging, those with vitreoretinal or corneal disease, or who had undergone vitreoretinal or corneal surgery in the past were not allowed to participate in the study. Patients with previous glaucoma treatment, including surgery, were not excluded. If severe surgery-related complications that could distort retinal imaging, such as a central choroidal detachment or persistent hyphema, were present at the first follow-up, patients were excluded from the study. 

In compliance with the guidelines of the World Glaucoma Association, all fellow eye surgeries were excluded from the database of this study [25].

All participants signed an informed consent form after having the study protocol thoroughly described to them, prior to enrolling. Patients were scheduled for re-visits at 1 month, 3 months, and 6 months after surgery. At baseline and each follow-up visit, all patients underwent a thorough ocular examination that included IOP assessment, refraction measures, slit lamp biomicroscopy, funduscopy, and OCTA imaging. At baseline, participants also received perimetric testing with the automated Humphrey visual field analyzer (HFA II, model 750, Carl Zeiss Meditec AG, Jena, Germany) with the standard program of the 30–2 Swedish interactive threshold algorithm (SITA fast). The occurrence of adverse events, as well as the frequency of postoperative 5-Fluorouracil (5-FU) injections were noted throughout the follow-up visits. 

### 2.3. Surgical Procedure

To ensure that there is no conjunctival hyperemia on the day of surgery, patients at our clinic discontinue using all glaucoma eye drops four weeks before Preserflo implantation. Instead, they are given corticosteroid eye drops three days before surgery and oral azetacolamide for four weeks. They then receive intravenous acetazolamide and mannitol 2–3 h before surgery in an effort to reduce the pre- to postoperative pressure gradient. The subsequent surgery for implanting the Preserflo Microshunt has been thoroughly described elsewhere [5,26,27]. In brief, mitomycin-C (MMC) 0.2 mg/mL is given to the bare sclera for 3 min, by inserting sponges into the conjunctival flap after the conjunctiva and Tenon’s capsule have been separated. A 2 mm deep scleral tunnel is formed using a 1 mm lance after rinsing with a balanced salt solution. A 25-gauge needle is then directed along this tract to enter the anterior chamber, producing a tunnel between the anterior chamber and the subconjunctival pocket located 3.5–4 mm from the limbus. The microshunt is retained inside the scleral pocket and introduced ab externo into the tunnel, with its tip reaching about 1–2 mm into the anterior chamber. Tenon’s capsule and conjunctiva are sealed after the device’s flow is verified by the appearance of drips at its outside end. 

### 2.4. OCTA Imaging

OCTA technology has been thoroughly explained elsewhere [11]. In brief, OCTA devices apply a very high scanning rate to capture images of retinal vessels. In contrast to static tissue, blood movement in retinal vessels causes variations in signal amplitude between subsequent B-scans [11]. On the basis of these differences, the internal decorrelation algorithm generates en face views of the retinal microvasculature for different retinal layers and regions. In the present study, both the macula and the peripapillary sectors were measured. For OCTA imaging, the spectral-domain (SD) RTVue XR Avanti system (Angiovue/RTVue-XR Avanti, Optovue Inc., Fremont, CA, USA) was employed. Eyes were imaged without topical dilatation. The macula was angiographically imaged using 3 mm^2^ scans, and papillary scans were performed using 4.5 mm^2^ images. Imaging was conducted using the device’s internal follow-up and tracking function to consistently map the same retinal region of each patient over the course of the study. The AngioVue algorithm automatically determined flow density (FD), which equals the ratio of bright pixels to the total number of pixels per scan, and is provided as a percentage value (%) for distinct retinal layers and sublocations.

OCTA imaging was conducted by an experienced examiner under identical circumstances for each patient and at the same site. Each papillary and macular slab underwent at least three consecutive images. The image with the greatest quality index (QI) among the three generated photos was chosen for study inclusion. If there were multiple photos with the same QI, one was randomly selected. Scans with artifacts or missing data were rejected. Images had to have a signal strength index (SSI) of 50.0 and a QI of 7. Manual sector segmentation was performed if necessary. 

**Figure 1:**
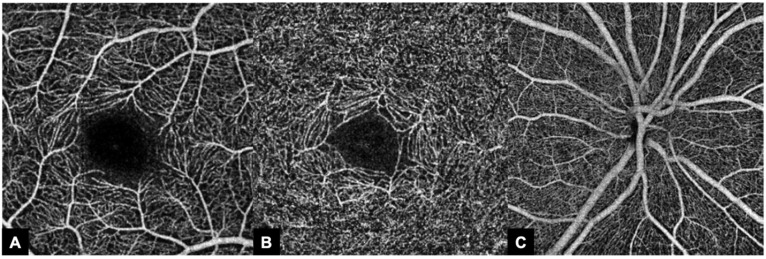
Representative en face angiograms of the regions analyzed using optical coherence tomography angiography (OCTA). (**A**): Superficial capillary plexus (SCP). (**B**): Deep capillary plexus (DCP). (**C**): (Peri)papillary capillary plexus with radial peripapillary capillaries (RPCs).

Each macular image contained FD data of the superficial (SCP) and deep macular plexus (DCP). In addition to these two, the FD values of the radial peripapilarry capillaries (RPCs) were extracted. Figure 1 depicts the OCT angiograms of the layers analyzed in the present study. 

The FD values for the whole en face images of the macular and peripapillary plexus, as well as IOP development before and after Preserflo MicroShunt implantation, were analyzed longitudinally. Statistical analysis investigated fluctuations in FD and IOP over the course of the entire follow-up period, comparing baseline values to distinctive follow-up intervals: one month, three months, and six months after surgery. Further subgroup analyses investigated changes in FD and IOP in accordance with disease severity. The latter was determined through perimetric testing on the basis of the Hodapp–Parrish–Anderson classification [28]. Our rationale for investigating disparities based on disease severity stems from the well-established correlation between visual field loss and the severity of glaucoma. Despite the complexities associated with the non-linear nature of these changes, our primary objective is to address the inquiry into vascular alterations concerning glaucoma severity.

### 2.5. Statistical Analysis

Statistical analysis was carried out in IBM SPSS Statistics, version 28.0. The Shapiro–Wilk test was used to assess the distribution of the data. The data did not follow a normal distribution. Therefore, IOP and OCTA measures were evaluated using Wilcoxon signed-rank test. For the statistical calculations of IOP and OCTA metrics, only patients with complete datasets were included. Since the study was exploratory, no adjustment for multiple testing was applied. All analyses are exploratory and should be considered as such. All *p-*values less than 0.05 were regarded as statistically significant.

## 3. Results

34 eyes from 34 patients were included in this study. All patients were pseudophakic at time of study inclusion. Further patient characteristics are summarized in Table 1. The median follow-up time was 115 (interquartile range (IQR): 33–226) days.

### 3.1. IOP Development

**Figure 2:**
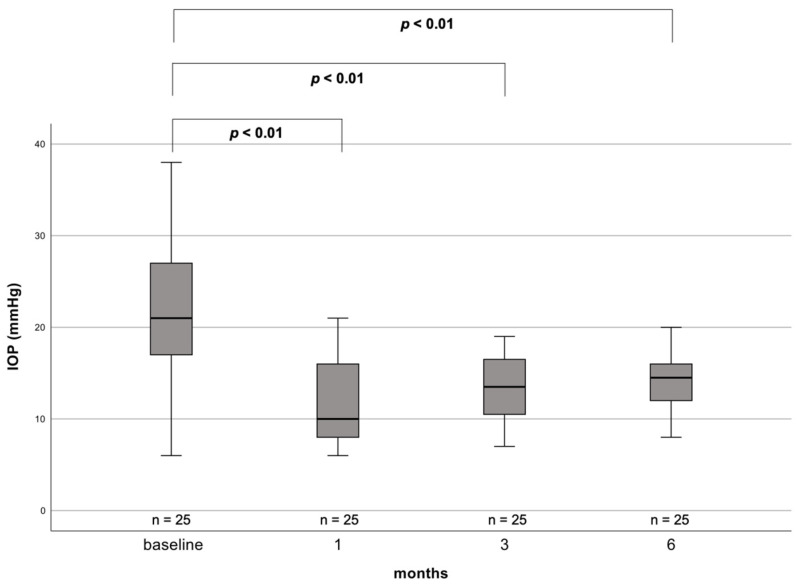
Boxplots showing IOP from baseline up to 6 months postoperatively for patients with complete datasets only. *p*-values for the difference between individual follow-up time points, as well as for the baseline-to-6-month comparison are presented and are derived from Wilcoxon signed-rank tests. *p*-values ≤ 0.05 are highlighted in bold. Note that distances between the time intervals are not to scale. IOP = intraocular pressure, mmHg = millimeters of mercury, *n* = number.

The median IOP reduction for the entire study cohort over the follow-up period was 8 (IQR 3–12) mmHg (*p* < 0.001; Figure 2, Table 2). Based on disease severity, IOP reduction was greatest in patients with advanced glaucoma, followed by patients with moderate and early glaucoma (Table 2). Due to the small sample size, caution is advised when interpreting IOP development data for moderate glaucoma eyes.

### 3.2. Postoperative Development

The median amount of supplemental medication dropped from 3 (IQR 3–4) at baseline to 1 (IQR 0–1) at follow-up (*p* < 0.01). 18 patients (53%) required supplemental medication after surgery and during the follow-up period. Of these, 11 patients (32%) administered carbonic anhydrase inhibitors, 6 (18%) took alpha agonists, and 1 (3%) continued therapy with carbonic anhydrase inhibitors and beta blockers.

Over the course of follow-up, 13 eyes (38%) were affected by hypotony (IOP ≤ 5  mmHg), with 6 eyes (18%) showing peripheral choroidal detachment. Central choroidal detachment was not observed in any patient. In all cases, hypotony resolved spontaneously, or with the support of topical steroid therapy during the first two weeks after surgery. 1 patient (3%) required revision surgery during the follow-up period to reposition the Preserflo Microshunt. 

### 3.3. Injections of 5-FU 

25 eyes (74%) received at least one subconjunctival injection of 5-FU during the follow-up period (range: 0–5; median: 2; interquartile range (IQR): 0–3).

### 3.4. Flow Density

**Figure 3:**
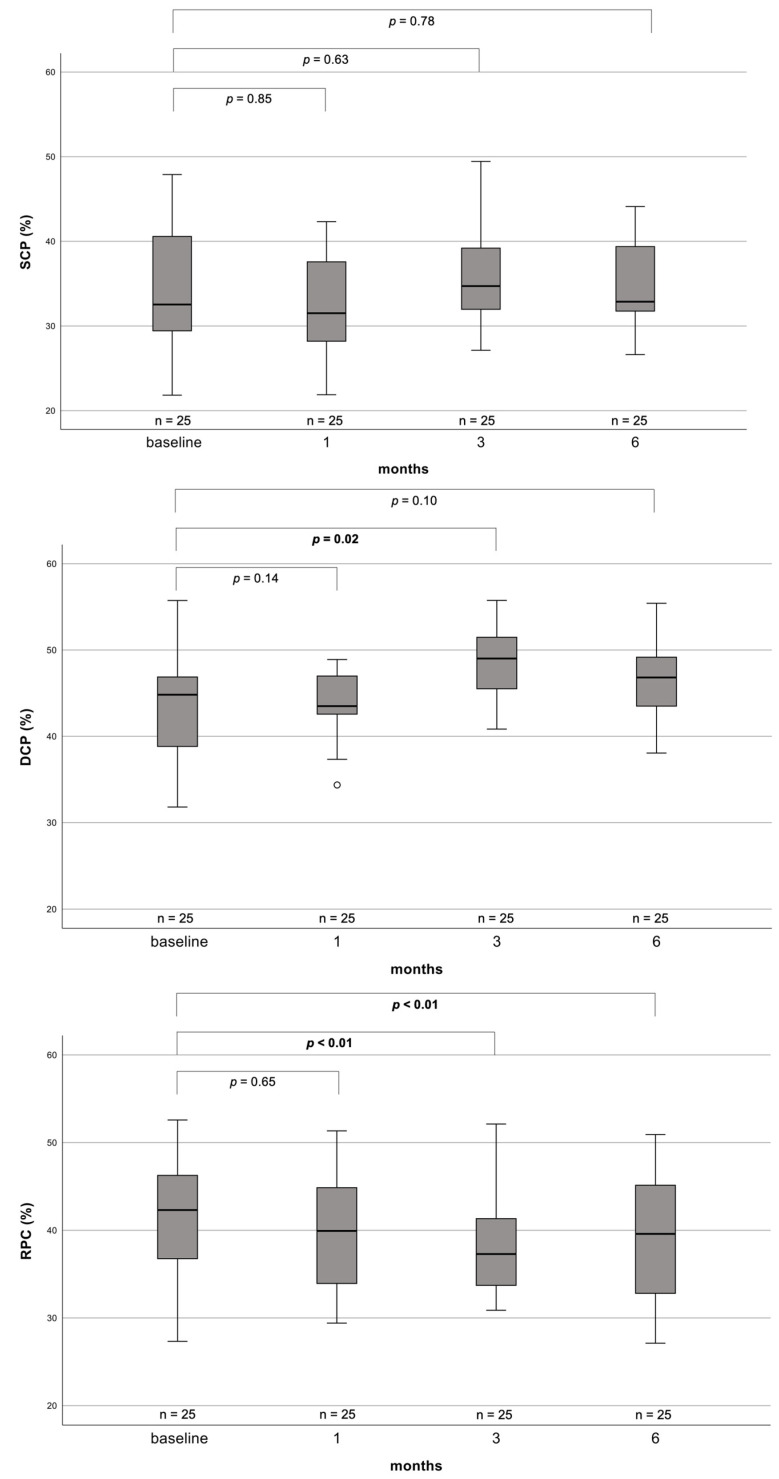
Boxplots depicting FD development from baseline up to 6 months postoperatively for patients with complete datasets only. *p*-values for the difference between baseline to individual follow-up time points are derived from Wilcoxon signed-rank tests. *p*-values ≤ 0.05 are highlighted in bold. Note that distances between the time intervals are not to scale. SCP = superficial capillary plexus, DCP = deep capillary plexus, RPC = radial peripapillary capillaries, *n* = number.

FD of the SCP decreased from 32.54 (IQR 21.10–43.98) at baseline to 32.87 (IQR 24.42–41.32) at six months postoperatively; however, this change was not statistically significant (*p* = 0.78). Similarly, while FD at the three-month interval was significantly increased in the DCP compared to baseline, there was no significant change in DCP FD between baseline (44.82; IQR 36.72–52.92) and the six-month time point (46.82; IQR 40.89–52.75) (*p* = 0.10). On the contrary, FD of the RPC changed significantly over the course of follow-up from 42.31 (IQR 32.65–51.97) at baseline, to 37.29 (IQR 27.09–47.49) at the three-month interval (*p* < 0.01), to 39.59 (IQR 25.72–53.46) after six months postoperatively (*p* < 0.01) (Figure 3). 

**Figure 4:**
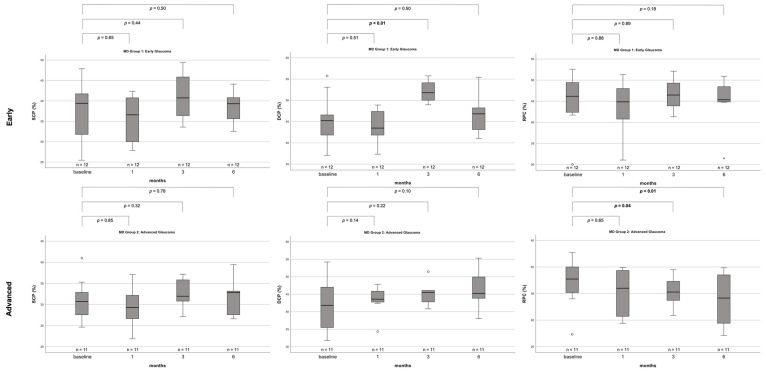
Boxplots depicting FD development from baseline up to 6 months postoperatively according to disease severity for patients with complete datasets only. Due to the small sample size, FD development for moderate glaucoma eyes is not displayed. *p*-values for the difference between baseline to individual follow-up time points are derived from Wilcoxon signed-rank tests. *p*-values ≤ 0.05 are highlighted in bold. Note that distances between the time intervals are not to scale. SCP = superficial capillary plexus, DCP = deep capillary plexus, RPC = radial peripapillary capillaries, *n* = number.

At the level of the SCP, the absence of substantial fluctuations in FD development remained, irrespective of disease severity. In the DCP of early glaucoma eyes, a significant increase in FD from baseline to month three with a subsequent reduction could be seen. The RPC showed a significant reduction of FD over the course of the entire follow-up period in eyes with advanced glaucoma, having a median FD of 37.73 (IQR 32.20–43.26) at baseline, which decreased to 34.15 (IQR 23.43–44.87) at six months postoperatively (*p* < 0.01). This was not seen in early disease eyes (baseline: 46.18; six months postoperatively: 45.34; *p* = 0.18) (Figure 4). Due to the small sample size, FD development for moderate glaucoma eyes is not displayed.

## 4. Discussion

The Preserflo MicroShunt has been shown to effectively lower IOP in glaucoma patients during long-term follow-up, irrespective of glaucoma subtype [5,6,7,29,30,31,32]. Recent studies have identified changes in retinal microvasculature related to IOP-lowering surgery in glaucoma patients [19,20,21,22,33]. This study represents the first trial to investigate changes in retinal FD in glaucoma patients related to treatment with the Preserflo MicroShunt.

In the present study, peripapillary FD in glaucoma eyes significantly decreased after the implantation of the Preserflo MicroShunt at the three- and six-month follow-up intervals. The reduction in FD was most apparent in eyes with advanced glaucomatous damage. FD of the macular layers, on the other hand, appeared less affected. Despite a temporary increase, FD of the DCP patients did not appear to be significantly altered at the latest follow-up in comparison to baseline, even when differentiating between disease severity groups. On the level of the SCP, no changes were detected throughout the follow-up period, even when differentiating between severity subgroups. 

The exact relationship between IOP fluctuations and OCTA metrics has yet to be fully understood. The majority of studies in this field of research have investigated the effect of trabeculectomy on retinal vascular architecture [20,21,33]. The focus on this treatment can be attributed to the fact that trabeculectomy, being the gold standard for penetrating glaucoma surgery, is firstly, widely used in the treatment of glaucoma and secondly, expected to cause significant and lasting reductions in IOP, thus increasing the likelihood of detecting possible changes in retinal FD related to alterations in IOP. Recently, however, a few other works have investigated changes in retinal FD in less invasive glaucoma treatments [19,34]. The present study contributes to this area of expertise by providing the first data on the effect of the Preserflo MicroShunt, as one of the representatives of LIGS procedures.

The results from the current literature on microvascular changes after IOP-lowering interventions are as diverse as the treatment options for glaucoma. While some authors have attributed changes in FD to IOP fluctuations [34,35], others found no relation between IOP and changes in FD [16,36,37]. Some have described an increase in FD in certain retinal layers after IOP-lowering surgery [19,20,21,22,33], while others have contrarily described a decreasing effect or no effect at all [23,24,38,39]. The field of OCT angiographic research has, all in all, yielded non-unanimous results in clinical settings. It is, however, important to note that comparing trials investigating the effect of IOP reduction on FD is challenging, as different trials may apply different inclusion criteria, surgical techniques, and OCTA devices. Aside from clinical trials, an experimental approach by Patel et al. investigated the effect of big IOP changes on OCTA metrics. Patel et al. [40] evaluated the effect of IOP variations on retinal FD in six healthy Macaca mulatta (rhesus monkey) eyes. Every 10 min, they experimentally increased IOP by 10 mmHg, going up to a maximum of 60 mmHg before lowering it again. Each time they reached a 10 mmHg increment, OCTA imaging was conducted. Only when IOP reached 50 mmHg did the authors notice a statistically significant decline in FD, with a progressive restoration to baseline when IOP was once more lowered to 10 mmHg. This work strongly suggests a reciprocal relationship between IOP and retinal FD. At the same time, it implies that noticeable changes in FD only emerge at high IOP levels. This rationale explains the increase in FD after IOP-lowering interventions noticed by several authors [19,20,33,34], and it may also explain why others have not observed such trends. In these cases, the reduction in IOP may simply not have been big enough to cause significant changes in FD. 

In the present study, retinal FD around the optic nerve head decreased significantly only in patients with advanced glaucoma. Eyes with early disease did not experience such changes. This observation could be related to differences in the amount of absolute IOP reduction in those subgroups. The IOP reduction over 6 months was smaller in early glaucoma eyes than in advanced glaucoma eyes, which could, in turn, translate into a lesser impact of IOP on FD in the early glaucoma subgroup. However, the findings by Patel et al. suggest that the differences in IOP reduction seen in this study between individual severity subgroups would most likely not be sufficient enough to cause a significant difference between these groups. On the other hand, they might lead to an increase in FD rather than a decrease. This suggests that the mere differences in IOP do not sufficiently explain the observations made in the present study. We believe that the changes observed can be better explained through the vascular autoregulatory mechanisms of the retina, which have been shown to dysfunction in glaucoma patients [41,42,43]. The findings of this trial fit with the current understanding of autoregulatory mechanisms in retinal perfusion, which sees a negative correlation between glaucoma severity and ocular blood flow [44]: eyes with early glaucomatous damage can still cope with moderate changes in IOP; however, advanced glaucomatous damage is associated with a reduced autoregulatory response to changes in IOP, which in this study, counterintuitively resulted in a reduction in FD around the optic disc. It can be postulated that we did not observe an increase in FD either because the deficient autoregulatory mechanisms in advanced glaucoma have led to a paradox reduction in FD in those eyes, or because autoregulatory mechanisms are delayed in advanced glaucoma, which would mean that a longer follow-up period could possibly reveal a postponed increase in FD. 

While the question of the exact influence of glaucoma surgery on retinal microvasculature remains part of an ongoing discussion, the present study presents new data on the influence of a moderate IOP decrease on retinal FD. Belonging to LIGS, the reduction in IOP caused by the Preserflo MicroShunt is expected to be less than what would otherwise be anticipated from classical penetrating glaucoma surgery, such as trabeculectomy [3]. In this trial, the Preserflo MichroShunt achieved a median decrease in IOP of 8 (IQR 3–12) mmHg over a follow-up period of 6 months. This amount is comparable to previous studies [5,6,7]. The comparably short follow-up duration of only 6 months should, however, be taken into account when interpreting IOP development in the present analysis. Typically, IOP reduction is greatest directly after surgery, followed by a consecutive increase in IOP to a level that is lower than it used to be at baseline. This development was also seen in the analysis of the present study cohort and should be considered when comparing the results of this study to other IOP-lowering interventions.

The quantitative assessment of longitudinal changes in retinal microvascular parameters subsequent to Preserflo MicroShunt implantation yields valuable insights with potential implications for clinical practice. The identified microvascular alterations might function as potential biomarkers, providing critical information for predicting outcomes post-Preserflo implantation. Notably, the observed impact of surgery on the RPCs in patients with advanced glaucomatous damage suggests that preoperative analysis of this specific vascular layer could aid in decision-making, guiding the selection of appropriate candidates for Preserflo implantation. Considering instances in which the FD of the RPC is already low before surgery, caution against the risk of further reduction might be advised. In such cases, alternative IOP-modifying therapies could turn out to be more beneficial. Additionally, our findings contribute to a broader understanding of microvascular dynamics in less invasive glaucoma treatments, serving as a foundational basis for further research.

### Limitations

This study has limitations, notably the lack of explicit assessment of ocular perfusion pressure and reliance on visual field grading for glaucoma severity. This hinders our ability to directly comment on autoregulatory mechanisms, introducing uncertainty in characterizing individual patients’ vascular responses. In addition, although the design of this study minimizes the impact of differences in baseline characteristics between individuals on its outcomes by assessing intra-individual development of IOP and FD, possible confounding effects of baseline vessel diameter, as well as magnification errors, should be considered.

As recent studies have demonstrated, IOP is not expected to change significantly six months after Preserflo MicroShunt implantation [5,6,7,29]. Therefore, changes in retinal OCTA metrics related to sudden changes in IOP after the latest follow-up are not expected in this study population either. Yet, there have been reports describing late changes in retinal FD occurring beyond the follow-up duration of six months [22]. We therefore cannot fully rule out that further changes in retinal microvasculature in this study population might become visible in the future. 

It is important to note that 53% of study participants required supplemental IOP-lowering medication after Preserflo surgery. These medications might have influenced FD values at the postoperative visits, as some studies demonstrated effects of antiglaucomatous medication on ocular blood flow [45,46,47]. 

In addition, this study only included patients with POAG and PEX glaucoma. Other glaucoma subtypes, such as normal-tension glaucoma, pigment dispersion glaucoma, or secondary glaucoma might yield different results, especially considering the fact that the exact mechanism between IOP fluctuations and FD alterations is not yet fully understood. 

Further prospective studies with larger patient cohorts and longer follow-up periods investigating the effect of Preserflo MicroShunt implantation on retinal FD are needed to validate the results presented in this trial and elaborate on late effects of this treatment on OCTA metrics.

## 5. Conclusions

To summarize, while peripapillary FD in glaucoma eyes decreased significantly following Preserflo MicroShunt implantation, FD in the macular layers appeared to be less affected. While the FD of the RPCs for the entire population over six months saw a median reduction of −2.72, the decrease was greater in patients with advanced glaucoma (−3.58) than in patients with early glaucoma (−0.84). This observation might hint towards reduced autoregulatory responses in capillaries surrounding the optic nerve head in comparison to their macular counterparts—especially in eyes with an advanced disease state. It is still unclear whether these changes in FD ought to be considered a result of or a cause of glaucoma progression.

## Figures and Tables

**Table 1:** General patient characteristics.

Characteristics	Study Cohort
Eyes—*n*	34
Patients—*n*	34
Age (years)—median	67 (61–73)
Gender (M:F)—*n* (%)	17 (50%):17 (50%)
Study eye (R:L)—*n* (%)	16 (47%):18 (53%)
Disease severity groups—*n* (%)	
early	16 (47%)
moderate	3 (9%)
advanced	15 (44%)
Type of glaucoma—*n* (%)	
POAG	26 (76%)
PEX glaucoma	8 (24%)
Previous surgery—*n* (%)	
none	19 (56%)
total	15 (44%)
SLT	10 (29%)
SLT + MIGS	3 (9%)
TE	2 (6%)
Spherical equivalent	−1.63 (−2.38–0.38)
Visual acuity (LogMar)	0.2 (0.3–0)
Arterial hypertension—*n* (%)	12
treated—*n* (%)	10
Diabetes mellitus—*n* (%)	2
treated—*n* (%)	2

Data are presented as median (25–75% interquartile range), or as absolute and relative values. Abbreviations: *n* = number, % = percentage, M = male; F = female, R = right, L = left, POAG = primary open angle glaucoma, PEX = pseudoexfoliation, LogMar = logarithm of the minimum angle of resolution, SLT = selective laser trabeculoplasty, MIGS = microinvasive glaucoma surgery, TE = trabeculectomy.

**Table 2:** IOP reduction at 6 months postoperatively in comparison to baseline for the entire patient population and by disease severity subgroups.

	IOP Baseline	IOP 6 Months	IOP Reduction
Total study population (mmHg)	*n* = 34	*n* = 25	*n* = 25
	22 (18–28)	15 (11–16)	8 (3–12)
Disease severity groups			
early (mmHg)	*n* = 16	*n* = 12	*n* = 12 6 (3–7)
	22 (18–25)	14 (9–16)	
moderate (mmHg)	*n* = 3	*n* = 2	*n* = 2 7 (6–7)
	21 (19–25)	14 (13–15)	
advanced (mmHg)	*n* = 15	*n* = 11	*n* = 11
	22 (18–27)	12 (9–15)	11 (6–14)

Data are presented as absolute numbers and median (25–75% IQR). Abbreviations: mmHg = millimeter mercury.

## Data Availability

Data are contained within the article.
